# 

**Published:** 1844-03

**Authors:** 


*To Correspondents.*—We regret that Dr. Thackston's very interesting Essay was received too late for insertion in the present number of the Journal. It shall, however, appear in our next, and we hope he will continue to furnish us with contributions from his able pen.

